# Physical Activity and Bullying Participant Roles Among South Korean Adolescents: Parallel Statistical Indirect Associations Through Psychosocial Resources

**DOI:** 10.3390/bs16071192

**Published:** 2026-07-15

**Authors:** Haoliang Wang, Woogyeon Jo

**Affiliations:** 1 Department of Physical Education, Graduate School, Kookmin University, Seoul 02707, Republic of Korea; whl534527524@gmail.com; 2 Department of Sports Industry & Leisure, Kookmin University, Seoul 02707, Republic of Korea

**Keywords:** physical activity, bullying participant roles, school bullying prevention, self-esteem, peer relationships, cooperativeness, adolescents

## Abstract

This cross-sectional secondary analysis examined concurrent associations between adolescents’ physical activity and five bullying participant roles and whether self-esteem, peer relationships, and cooperativeness statistically accounted for these associations. Data collected in 2023 from 2242 ninth-grade participants in the sixth wave of the Korean Children and Youth Panel Survey 2018 were analyzed using correlations and five outcome-specific PROCESS Model 4 analyses, controlling for gender. Physical activity was positively associated with self-esteem, peer relationships, and cooperativeness (*B* = 0.031–0.070, all *p* < 0.001). Small negative indirect associations with direct bullying, assisting the bully, and reinforcing the bully were observed through peer relationships (indirect estimates = −0.023 to −0.019) and cooperativeness (indirect estimates = −0.003 to −0.002). A small positive indirect association with defending the victim was observed through self-esteem (indirect estimate = 0.007), whereas no indirect association was found for avoidance. Although several associations were statistically detectable, their magnitudes and overall explanatory power were limited. Because all variables were measured concurrently, these findings do not establish temporal or causal mediation. They may inform hypotheses and the design of future longitudinal evaluations of cooperative, relationship-supportive physical-activity contexts, but they do not demonstrate that physical-activity or sport programs reduce bullying.

## 1. Introduction

School bullying remains a major educational and social concern because it is associated with emotional distress, school maladjustment, reduced belonging, and unsafe learning environments. International reports continue to identify bullying and peer victimization as threats to students’ well-being and participation in school life (UNESCO, 2019; OECD, 2023). In South Korea, school violence is monitored through a national survey and includes verbal aggression, physical violence, and group exclusion, underscoring the continuing policy relevance of peer-group processes in Korean schools (Ministry of Education, 2023).

Bullying is not only a dyadic interaction between a perpetrator and a victim; it is a group process in which peers can initiate, assist, reward, oppose, or avoid the behavior. The participant-role framework distinguishes direct bullies, assistants, reinforcers, defenders, and outsiders or avoidant bystanders (Salmivalli et al., 1996; Salmivalli & Voeten, 2004). More recent participant-role research similarly emphasizes that bystander roles are behaviorally distinct and are shaped by peer pressure, group norms, and social positioning (Xie & Ngai, 2020).

These roles should not be treated as interchangeable. Direct bullying involves initiating or leading aggression and may be especially related to individual regulation and moral disengagement. Assisting involves joining or materially helping an aggressor, whereas reinforcing involves rewarding bullying through attention, laughter, or encouragement; both are likely to be sensitive to conformity and peer-group norms. Defending requires active intervention on behalf of the victim and may entail social risk, while avoidance reflects non-involvement and may arise from fear, uncertainty, or situational judgment rather than simple indifference. Longitudinal evidence linking moral disengagement with bullying supports the need to distinguish mechanisms that sustain active aggression from those associated with defending or passive bystanding (Thornberg, 2023).

Physical activity is associated with a range of adolescent health and psychosocial indicators, although the strength and direction of associations vary by outcome, activity context, and study design. Systematic reviews and meta-analyses report generally favorable but often modest associations with mental health and psychosocial well-being (Poitras et al., 2016; Rodriguez-Ayllon et al., 2019; Biddle et al., 2019). A mechanism-focused review also identifies self-perception and social connectedness as plausible pathways (Lubans et al., 2016). Longitudinal work also suggests that social connectedness and self-esteem may be among the mechanisms linking physical activity with mental health (Doré et al., 2020). A recent systematic review and best-evidence synthesis further identified self-esteem, social support, and social connection among the more consistently supported psychosocial mechanisms linking physical activity with mental-health outcomes, while also emphasizing substantial heterogeneity across populations, outcomes, and study designs (White et al., 2024). At the same time, a recent meta-analysis found a negative association between physical activity and bullying victimization but no clear overall association with bullying perpetration, indicating that links between activity and bullying are heterogeneous rather than uniformly protective (Liu et al., 2024).

The present model draws on complementary theoretical perspectives. The social development model proposes that opportunities for involvement, skills, reinforcement, and bonding can shape adherence to prosocial or antisocial norms (Catalano & Hawkins, 1996). Self-determination theory highlights competence and relatedness as basic psychological needs that can be supported or frustrated within activity settings (Ryan & Deci, 2017). Moral disengagement research further indicates that cognitive justifications and diffusion of responsibility can facilitate bullying behavior (Thornberg, 2023). Accordingly, physical activity may be associated with bullying roles not simply because movement is beneficial, but because some activity settings provide opportunities for competence, peer bonding, rule-governed cooperation, and prosocial recognition. These mechanisms depend on how activities are organized; competitive, exclusionary, or poorly supervised contexts may not generate the same psychosocial resources as inclusive and cooperative contexts.

Self-esteem, peer relationships, and cooperativeness were therefore examined as distinct, concurrent psychosocial resources. Self-esteem represents an intrapersonal evaluation that may be relevant to socially risky defending behavior. Peer relationships represent a relational resource, but their association with bullying may depend on whether prevailing group norms are prosocial or pro-bullying. Cooperativeness represents a behavioral capacity to coordinate roles, pursue shared goals, and consider others, and may be especially relevant to assisting and reinforcing behaviors that depend on group participation. Modeling these variables in parallel allows their unique statistical associations to be estimated without imposing an unsupported sequential order among them.

Despite growing evidence linking physical activity with psychosocial well-being and bullying-related outcomes, previous research has often focused on broad categories of bullying perpetration and victimization rather than on the differentiated participant roles that sustain, oppose, or avoid bullying situations. Consequently, comparatively less is known about whether physical activity shows similar or different associations with direct bullying, assisting, reinforcing, defending, and avoidance, and whether distinct intrapersonal, relational, and behavioral resources statistically account for these role-specific patterns. Examining the five roles within a common analytical framework may therefore provide a more differentiated understanding of the concurrent associations between physical activity and adolescents’ behavior in bullying situations.

The South Korean context is substantively relevant because bullying is addressed as a school-wide peer-group issue through national monitoring, while adolescents’ peer relationships and physical activity occur largely within structured school and extracurricular environments. The focus on Grade 9 was also deliberate. In South Korea, Grade 9 is the final year of middle school and immediately precedes the transition to high school, a period in which students prepare for and anticipate changes in school environments and peer networks while facing heightened academic expectations. Korean longitudinal evidence indicates that school adjustment changes across this transition and that peer and teacher relationships are associated with its trajectory (Cho & Lee, 2008). Grade 9 is therefore a meaningful developmental and educational period in which peer relationships, self-evaluation, and emotional adjustment may be especially salient to bullying participant roles. However, cultural and institutional differences in classroom norms, teacher intervention, competitive climates, and access to organized physical activity may shape the meanings of both physical activity and bullying participant roles. Accordingly, the observed associations should not be assumed to generalize directly to other educational systems.

Two qualifications are important at the outset. First, the KCYPS physical-activity item captures only the reported amount of recent exercise sufficient to induce sweating; it does not identify activity type, frequency pattern, instructional quality, team versus individual participation, or cooperative versus competitive structure. The theoretical discussion of activity context, therefore, provides a rationale for future research rather than a direct measurement of those qualities in this study. Second, all focal variables were measured concurrently. The analysis estimates cross-sectional associations and statistical indirect associations, not temporal or causal mediation. Reverse or reciprocal patterns are plausible: adolescents with more positive peer adjustment may select into physical activity, while peer-group status and bullying involvement may also facilitate or constrain participation.

The present study aimed to examine the concurrent associations between self-reported physical activity and five bullying participant roles among South Korean ninth-grade adolescents and to determine whether self-esteem, peer relationships, and cooperativeness statistically accounted for these associations. Specifically, the study (1) described the bivariate relationships among physical activity, the three psychosocial resources, and the five participant roles; (2) estimated the gender-adjusted concurrent associations of physical activity with self-esteem, peer relationships, and cooperativeness; and (3) examined role-specific direct and parallel statistical indirect associations between physical activity and bullying participant roles. By distinguishing the five roles, the study sought to determine whether the observed associations followed a broadly uniform pattern across bullying situations or differed according to the behavioral and social characteristics of each participant role.

Based on the participant-role framework and the literature reviewed above, the following directional hypotheses were specified:

**Hypothesis 1** **(H1).** *Physical activity will be positively associated with self-esteem, positive peer relationships, and cooperativeness.*

**Hypothesis 2** **(H2).** *Physical activity will be negatively associated with direct bullying, assisting the bully, and reinforcing the bully, and positively associated with defending the victim.*

**Hypothesis 3** **(H3).** *More positive peer relationships and greater cooperativeness will be negatively associated with pro-bullying roles, whereas self-esteem will be positively associated with defending the victim.*

**Hypothesis 4** **(H4).** *Physical activity will show negative statistical indirect associations with pro-bullying roles through peer relationships and cooperativeness and a positive statistical indirect association with defending the victim through self-esteem.*

Because avoidance of the bullying situation may reflect heterogeneous motives, including fear, uncertainty, situational absence, or deliberate non-involvement, no directional hypothesis was formulated for this role. Instead, the following exploratory question was examined:

Exploratory Research Question. What direct and statistical indirect associations are observed between physical activity and avoidance of the bullying situation?

## 2. Materials and Methods

### 2.1. Data and Participants

This study was a cross-sectional secondary analysis of the Korean Children and Youth Panel Survey 2018 (KCYPS 2018), conducted by the National Youth Policy Institute. The KCYPS 2018 established two original panels in 2018: 2607 fourth-grade elementary school students and 2590 first-year middle school students. Data were collected using Tablet-Assisted Personal Interviewing (TAPI), a tablet-based individual interview method. Although the KCYPS is longitudinal, the present analysis used one wave and should therefore be interpreted as cross-sectional.

The sixth wave of the fourth-grade cohort, collected in 2023 when participants were in Grade 9, was selected for both substantive and measurement reasons. Substantively, Grade 9 is the final year of middle school in South Korea and immediately precedes entry into high school. This pre-transition period was considered especially relevant because students are preparing for and anticipating changes in school environments and peer networks while facing heightened academic expectations, which may make peer relationships and emotional adjustment particularly salient. Methodologically, Wave 6 concurrently included the participant-role items and all focal psychosocial variables required for the prespecified role-comparison model. Using one wave, therefore, enabled a consistent comparison of five role outcomes at the same developmentally meaningful stage, but it did not establish temporal ordering. The final analytic sample consisted of 2242 adolescents with valid data on physical activity, self-esteem, peer relationships, cooperativeness, the five bullying participant roles, and gender.

The sixth wave of the KCYPS 2018 was conducted after review and approval by the Institutional Review Board of the National Youth Policy Institute (approval no. NYPI-202306-HR-041-01; June 2023). Survey information, consent procedures, and personal-information protections were provided during data collection. The present study used publicly available, fully de-identified secondary data supplied by the National Youth Policy Institute and contained no personally identifiable information.

### 2.2. Measures

The focal variables were constructed from sixth-wave adolescent-response items. Physical activity was the exposure variable; the five bullying participant roles were analyzed as separate outcomes; and self-esteem, peer relationships, and cooperativeness were entered in parallel in the statistical indirect-association models. Gender was included as an a priori covariate. Mean scores were calculated for multi-item scales, and higher scores indicated higher levels of the corresponding construct or role behavior.

#### 2.2.1. Physical Activity

Physical activity was assessed with one item from the health and physical development domain of the KCYPS 2018 questionnaire (National Youth Policy Institute, 2024): “During the past week, about how many hours did you exercise enough to sweat?” Responses were coded 1 = none, 2 = 1 h, 3 = 2 h, 4 = 3 h, and 5 = 4 h or more. This item was retained because it was the available common physical-activity indicator in the secondary dataset and provided a brief ordinal proxy for recent sweat-inducing exercise volume. It should not be interpreted as a comprehensive or psychometrically validated measure of habitual physical activity: it does not distinguish frequency, precise intensity, activity type, participation context, or individual versus team sport, and internal-consistency reliability is not applicable to a single item (Sallis & Saelens, 2000).

#### 2.2.2. Self-Esteem

Self-esteem was measured using items from the self-esteem scale in the social, emotional, and competency development domain of the KCYPS 2018 questionnaire (National Youth Policy Institute, 2024). These items were based on the original Rosenberg Self-Esteem Scale developed by Rosenberg (1965), which was adapted and used in the KCYPS 2010 and subsequently revised for the KCYPS 2018. The scale consisted of 10 items, including positive items such as “I am satisfied with myself,” “I feel that I have many strengths,” “I feel that I am a person of worth, at least on an equal basis with others,” and “I have a positive attitude toward myself,” as well as negative items such as “At times, I think I am no good at all” and “I tend to feel that I am a failure.” Each item was rated on a four-point Likert scale ranging from 1 = strongly disagree to 4 = strongly agree. Negative items were reverse-coded, and the mean score was calculated. Higher scores indicated higher levels of self-esteem. In the present sample, internal consistency was acceptable (Cronbach’s *α* = 0.792).

#### 2.2.3. Peer Relationships

Peer relationships were measured using items from the peer relationship scale in the school and peer relationship domain of the KCYPS 2018 questionnaire (National Youth Policy Institute, 2024). This scale consisted of 13 items based on the Adolescent Peer Relationship Quality Scale developed and validated by Bae et al. (2015). Sample items included “I spend time with my friends,” “I talk openly about myself with my friends,” “I have good relationships with my friends,” “I often have disagreements with my friends,” and “When I have a conflict with a friend, we do not reconcile easily.” Each item was rated on a four-point Likert scale ranging from 1 = strongly disagree to 4 = strongly agree. In this study, negatively worded items were reverse-coded so that higher scores represented more positive peer relationships, and the mean score was used in the analysis. In the present sample, internal consistency was good (Cronbach’s *α* = 0.829).

#### 2.2.4. Cooperativeness

Cooperativeness was measured using items from the cooperation subdomain of the social, emotional, and competency development domain of the KCYPS 2018 questionnaire (National Youth Policy Institute, 2024). The scale consisted of 14 items adapted from the cooperation domain of the youth social participation competency measure developed by Kim et al. (2015). Sample items included “I actively acknowledge my friends’ strengths or abilities,” “I willingly help my friends when they are in difficulty,” “I do my best in my assigned role,” and “When important issues or problems arise, I solve them together with my friends.” Each item was rated on a four-point Likert scale ranging from 1 = strongly disagree to 4 = strongly agree. Because all items were positively worded, the mean score was calculated without reverse-coding. Higher scores indicated higher levels of cooperativeness. In the present sample, internal consistency was excellent (Cronbach’s *α* = 0.930).

#### 2.2.5. Roles in School Bullying

Bullying participant roles were assessed with 15 KCYPS items adapted from the short form of the Participant Role Questionnaire and organized into five prespecified three-item subscales: direct bullying, assisting the bully, reinforcing the bully, defending the victim, and avoidance of the bullying situation (Salmivalli et al., 1996; Salmivalli & Voeten, 2004; National Youth Policy Institute, 2024). Items were rated from 1 = never to 4 = often, and the mean of the three items was calculated for each role. The exact item wording and response scale are provided in Supplementary Table S1 to improve transparency and reproducibility.

Direct bullying captures initiating or organizing aggression; assisting captures joining or materially helping the bully; reinforcing captures attention, laughter, or verbal encouragement that rewards bullying; defending captures comforting the victim or actively attempting to stop the behavior; and avoidance captures staying away from or declining involvement in the situation. Internal consistency in the present sample was *α* = 0.837 for direct bullying, 0.865 for assisting, 0.808 for reinforcing, 0.857 for defending, and 0.886 for avoidance.

The five role scores were analyzed separately because they represent behaviorally and theoretically distinct positions rather than a single continuum. The study retained the five-subscale structure documented by the KCYPS and evaluated the internal consistency of each subscale; however, the five-factor structure was not independently examined through confirmatory factor analysis in the present sample.

#### 2.2.6. Control Variable

Gender was selected a priori as the sole covariate because it is conceptually relevant to both adolescent physical activity and bullying participant roles. The original code (1 = male, 2 = female) was recoded as 0 = male and 1 = female, with males as the reference group. Urbanicity was retained in Table 1 to describe the distribution of school locations in the analytic sample but was not included in the prespecified inferential model. Urbanicity was treated as a broad sample descriptor rather than an adequate proxy for individual socioeconomic circumstances, neighborhood conditions, or school climate. Other potentially relevant variables, including socioeconomic conditions, academic indicators, school climate, parental involvement, and baseline mental health, were not modeled. Consequently, residual confounding and omitted-variable bias cannot be excluded.

### 2.3. Data Analysis

Data were analyzed using IBM SPSS Statistics version 25.0 and PROCESS macro version 4.2 (Hayes, 2022). Item direction was reviewed before scale construction. Negatively worded self-esteem and peer-relationship items were reverse-coded; cooperativeness and participant-role items were scored in their original directions. Descriptive statistics, Cronbach’s *α* for multi-item scales, and Pearson correlations were calculated. Multicollinearity was assessed in each of the five role-specific outcome equations using tolerance and variance inflation factor (VIF) values for gender, physical activity, self-esteem, peer relationships, and cooperativeness.

The three psychosocial variables were modeled in parallel because they were conceptualized as distinct resources—intrapersonal, relational, and behavioral—and the concurrent design did not support a defensible temporal sequence among them. Five role-specific PROCESS Model 4 analyses were estimated so that direct bullying, assisting, reinforcing, defending, and avoidance were not collapsed into a conceptually heterogeneous total score. Physical activity was entered as the exposure, the three psychosocial resources were entered in parallel in the statistical indirect-association models, and gender was entered as a covariate in all psychosocial-variable and outcome equations. These models estimate conditional associations and products of coefficients; the term “indirect association” is used to avoid implying temporal or causal mediation.

Because the same sample was used for five correlated outcomes, the analyses entail multiplicity and do not model residual covariances among outcomes as an integrated structural equation model would. The role-specific results were therefore interpreted cautiously as a coherent overall pattern rather than as five independent definitive findings. Correlations among the psychosocial variables were also considered when interpreting their unique coefficients; no causal or developmental ordering among these variables was assumed.

Statistical indirect associations were evaluated with 5000 bootstrap samples and 95% bootstrap confidence intervals; intervals excluding zero were treated as statistically detectable (Preacher & Hayes, 2008). The reported *p* values and bootstrap confidence intervals were not adjusted for multiplicity. The three pro-bullying outcomes were retained on their original 1–4 subscale-mean metric to preserve interpretability and comparability across participant roles; no transformation was applied. Their positive skewness was treated as a substantive feature of low-frequency pro-bullying behavior in a general school sample rather than as a condition automatically requiring transformation. Bootstrapping was used specifically to estimate confidence intervals for products of coefficients and was not treated as a remedy for outcome skewness, possible non-normal residuals, or heteroscedasticity. Because all focal variables were reported by the same adolescents at the same time, common method variance remains possible (Podsakoff et al., 2003). As a supplementary assessment of common method variance, Harman’s single-factor test was conducted by entering all 53 self-reported items into an unrotated principal component analysis. The number of components with eigenvalues greater than 1 and the proportion of variance explained by the first component were examined. No integrated structural equation model was estimated to model the five correlated role outcomes simultaneously.

## 3. Results

### 3.1. Descriptive Statistics, Normality, and Reliability

The results of the descriptive statistics, normality, and reliability analyses are presented in Table 2. The psychosocial variables—self-esteem, peer relationships, and cooperativeness—were all above the midpoint of the four-point scale. Regarding the subdimensions of roles in school bullying, the pro-bullying roles, including direct bullying, assisting the bully, and reinforcing the bully, showed relatively low mean scores. In contrast, defending the victim and avoidance of the bullying situation showed relatively higher mean scores.

The three pro-bullying outcomes showed positive skewness ranging from 2.148 to 2.324 and kurtosis ranging from 4.438 to 5.251, consistent with low self-reported involvement in a general school sample. Although these values were below conventional heuristic thresholds (Kline, 2011), such thresholds do not establish normal residuals or homoscedasticity. Accordingly, findings for these low-frequency outcomes are interpreted cautiously, and the bootstrap confidence intervals for products of coefficients should not be taken as evidence that all distributional concerns in the outcome equations were removed.

Cronbach’s *α* values ranged from 0.792 to 0.930 across all multi-item scales, indicating acceptable to excellent internal consistency. Reliability was not calculated for the single physical-activity item.

As a supplementary diagnostic of common method variance, Harman’s single-factor test did not identify a dominant single factor. The Kaiser–Meyer–Olkin measure was 0.938, and Bartlett’s test of sphericity was significant, *χ*^2^(1378) = 62,083.007, *p* < 0.001. Eight components had eigenvalues greater than 1, and the first component accounted for 19.290% of the total variance. Thus, no single general factor accounted for the majority of the item variance.

### 3.2. Bivariate Correlations Among Study Variables

Table 3 presents the bivariate correlations. Physical activity was positively correlated with self-esteem, peer relationships, and cooperativeness, consistent with H1. The correlations were statistically significant but small in magnitude.

Physical activity was negatively correlated with direct bullying, assisting, and reinforcing and positively correlated with defending and avoidance. These coefficients describe concurrent bivariate associations and do not establish that physical activity preceded or changed any role behavior.

Self-esteem and peer relationships were negatively correlated with the three pro-bullying roles and positively correlated with defending. Cooperativeness was negatively correlated with direct bullying, assisting, and reinforcing but was not significantly correlated with defending or avoidance. The psychosocial variables were themselves correlated, particularly self-esteem and peer relationships (*r* = 0.456), so their coefficients in the parallel models represent unique associations conditional on the other psychosocial variables rather than independent developmental processes.

Gender was negatively correlated with physical activity. With gender coded 0 = male and 1 = female, female students reported lower physical-activity scores on average. Gender was therefore retained as the prespecified covariate.

### 3.3. Physical Activity and Psychosocial Resources

Controlling for gender, physical activity was positively associated with all three psychosocial variables (Table 4): peer relationships (*B* = 0.070, *β* = 0.230, *p* < 0.001), self-esteem (*B* = 0.057, *β* = 0.181, *p* < 0.001), and cooperativeness (*B* = 0.031, *β* = 0.082, *p* < 0.001). H1 was therefore supported. The standardized association with cooperativeness was small, and the models explained 0.6–5.5% of the variance in the psychosocial variables.

### 3.4. Adjusted Concurrent Associations with Bullying Participant Roles

Table 5 presents coefficients from the five role-specific models. Each coefficient is an adjusted concurrent association conditional on gender and the other variables in the model; it should not be interpreted as a prospective effect. Multicollinearity diagnostics indicated no substantial concern: tolerance values ranged from 0.743 to 0.942, and VIF values ranged from 1.062 to 1.347. Because the same predictor set and analytic sample were used across all five outcome models, the diagnostic values were identical. The coefficients for self-esteem, peer relationships, and cooperativeness therefore represent unique conditional associations after accounting for the other predictors in each parallel model.

For pro-bullying roles, physical activity was negatively associated with direct bullying and assisting, but its adjusted association with reinforcing did not reach conventional statistical significance (*p* = 0.059). Peer relationships and cooperativeness were negatively associated with all three pro-bullying roles, whereas self-esteem was not. Thus, H2 was partially supported, whereas H3 was supported.

For defending, physical activity and self-esteem showed positive adjusted associations, whereas peer relationships and cooperativeness did not. The model explained only 1.6% of the variance (*R*^2^ = 0.016), indicating limited practical explanatory value despite statistically detectable coefficients.

For avoidance, only physical activity showed a statistically detectable positive coefficient. The model explained 0.7% of the variance (*R*^2^ = 0.007); consequently, this result is treated as weak exploratory evidence and is not used to support substantive claims about the determinants of avoidance.

### 3.5. Bootstrap Tests of Statistical Indirect Associations

Table 6 reports products of coefficients and their 5000-sample bootstrap confidence intervals. These estimates are termed statistical indirect associations because the concurrent design does not establish temporal mediation.

For direct bullying, assisting, and reinforcing, the products through peer relationships were negative (indirect estimates = −0.023 to −0.019), as were the smaller products through cooperativeness (−0.003 to −0.002); their 95% bootstrap confidence intervals excluded zero. The products through self-esteem included zero. These patterns were consistent with the pro-bullying portion of H4, but their absolute magnitudes were small.

For defending, the product through self-esteem was positive and small (indirect estimate = 0.007, 95% bootstrap CI [0.002, 0.013]); products through peer relationships and cooperativeness included zero. This pattern was consistent with the defending portion of H4, but the low model *R*^2^ limits its practical interpretation. Taken together, these results supported H4.

For avoidance, all three bootstrap intervals included zero. The model therefore provided no evidence that the selected psychosocial variables statistically accounted for the small concurrent association between physical activity and avoidance.

## 4. Discussion

This cross-sectional study examined whether self-esteem, peer relationships, and cooperativeness statistically accounted for concurrent associations between physical activity and five bullying participant roles. H1 was supported: physical activity was positively associated with all three psychosocial resources. H2 was partially supported, whereas H3 and H4 were supported. Peer relationships and cooperativeness accounted for small negative associations with pro-bullying roles, while self-esteem accounted for a small positive association with defending. No indirect association was detected for avoidance. The results should be interpreted as role-specific correlational patterns, not as evidence that physical activity produces psychosocial change or reduces bullying. Importantly, the differentiated pattern across the five roles supports the value of examining bullying as a peer-group process involving behaviorally distinct forms of participation rather than as a single undifferentiated outcome.

### 4.1. Physical Activity and Psychosocial Resources

The positive associations with self-esteem and peer relationships are broadly consistent with contemporary reviews linking physical activity with mental-health and psychosocial indicators in youth and broader populations (Poitras et al., 2016; Rodriguez-Ayllon et al., 2019; Biddle et al., 2019; White et al., 2024). Doré et al. (2020) also identified social connectedness and self-esteem as plausible longitudinal mechanisms. However, the present coefficients were modest, and the evidence base does not support a universal psychosocial benefit across all activity contexts. School-related intervention reviews likewise show heterogeneity across program designs and outcomes (Andermo et al., 2020).

The association with peer relationships was stronger than that with cooperativeness, but this difference should not be interpreted as proof that activity builds relationships. Physical activity can create opportunities for relatedness, recognition, and shared tasks, as proposed by self-determination and social-development perspectives. When such opportunities are repeatedly available, participation in physical activity may co-occur with relational experiences that are relevant to adolescents’ integration and functioning within peer groups. The very small association with cooperativeness is particularly important: the amount of sweat-inducing exercise measured here cannot represent the cooperative structure, inclusion, supervision, or moral climate of an activity setting.

Accordingly, the findings support a limited statement: physical activity co-occurred with positive psychosocial resources in this sample. They do not identify which sports, instructional practices, or peer climates produced those associations. Future studies should distinguish team and individual activities, competitive and cooperative formats, school physical education and extracurricular sport, and supportive versus exclusionary climates. Thus, the present findings are consistent with the possibility that the psychosocial meaning of physical activity depends not only on how much activity occurs, but also on the relational and instructional qualities of the setting, although those qualities were not directly assessed.

### 4.2. Role-Specific Associations

The role-specific results reinforce the value of separating direct bullying, assisting, reinforcing, defending, and avoidance. Direct bullies initiate or organize aggression; assistants join active aggression; and reinforcers reward it through social attention. Peer relationships and cooperativeness were negatively associated with all three roles, but the possible mechanism may differ. For assistants and reinforcers, the pattern is compatible with peer-norm and conformity processes. For direct bullying, individual regulation, status motives, and moral disengagement may be more relevant. Despite these possible role-specific differences, the consistent negative associations of peer relationships and cooperativeness across all three pro-bullying roles are noteworthy because each role involves active participation in initiating, supporting, or socially rewarding aggression. The negative coefficient for peer relationships should not be interpreted as indicating that simply having more friends is protective. Rather, the quality of peer relationships and the norms prevailing within those relationships may be more relevant to participation in pro-bullying behavior (Salmivalli & Voeten, 2004). Because these mechanisms were not directly measured, these interpretations remain theoretical possibilities.

Self-esteem was not uniquely associated with the three pro-bullying roles but was positively associated with defending. Defending requires more than non-participation: it can involve confronting peers, supporting the victim, or seeking adult help despite possible social costs. A positive self-evaluation may therefore be more relevant to active defending than to the absence of aggression. Even so, the defending model explained only 1.6% of the variance, so the result should be treated as a small association requiring replication rather than a strong explanatory account.

Avoidance warrants still greater restraint. Although physical activity had a positive adjusted coefficient, none of the psychosocial variables accounted for that association, and the model explained only 0.7% of the variance. Avoidance may reflect fear of retaliation, uncertainty, lack of perceived efficacy, situational absence, or classroom norms, but the current model did not measure these explanations. Accordingly, this isolated finding is insufficient to support practical recommendations.

### 4.3. Statistical Indirect Associations and Alternative Explanations

The products of coefficients through peer relationships and cooperativeness were statistically detectable for the three pro-bullying roles, while the product through self-esteem was detectable for defending. These results align with the proposed distinction between relational or behavioral resources and intrapersonal resources. The recurrence of this pattern across all three pro-bullying roles suggests that relational and behavioral resources may be more consistently relevant to active support for bullying, whereas the intrapersonal resource represented by self-esteem may be more specifically relevant to active defending. However, the indirect estimates were small, and a product-of-coefficients result from concurrent data cannot demonstrate that physical activity occurred first, changed a psychosocial resource, and subsequently changed a bullying role.

Reverse, reciprocal, and confounded explanations remain plausible. Adolescents who are less aggressive, more cooperative, or better integrated with peers may be more likely to participate in physical activity, while peer-group status and norms may facilitate or constrain participation. Family resources, academic pressures, school climate, teacher support, and prior mental health may also contribute to the observed covariance. The present models, therefore, provide a basis for longitudinal hypotheses rather than evidence of causal mechanisms.

The small size of the indirect estimates is also important for substantive interpretation. Statistical significance was facilitated by the large sample, whereas practical importance depends on magnitude, replicability, and whether associations predict meaningful change over time. The recent meta-analytic finding that physical activity is related more consistently to victimization than to perpetration further cautions against assuming a general anti-bullying effect (Liu et al., 2024).

### 4.4. Cultural Context and Cautious Practical Implications

The South Korean setting provides an important boundary condition. National monitoring indicates that school violence remains a policy concern, and participant roles unfold within school-specific peer norms and intervention systems (Ministry of Education, 2023). The meaning of defending, remaining outside, or joining peers may differ across educational cultures, classroom structures, teacher practices, and opportunities for organized physical activity. The findings should therefore not be generalized automatically to Western or other school systems. Cross-national and multilevel studies are needed to test whether school and cultural contexts modify these associations.

The results do not show that physical education or sport programs reduce bullying. At most, they suggest design considerations that warrant prospective testing. Schools and physical educators could evaluate structured activities that emphasize inclusive grouping, rotating roles, shared goals, peer support, non-humiliating feedback, and safe opportunities to seek adult help. These components are theoretically more relevant to peer relationships, cooperativeness, and defending than simply increasing activity time. For defending-related outcomes, prospective programs could also examine whether opportunities for successful role performance, responsible leadership, peer recognition, and safe help-seeking support students’ confidence to intervene. Competitive formats should not be presumed beneficial unless their social climate and supervision are also addressed.

Any implementation should be integrated with established school-wide anti-bullying procedures rather than presented as a stand-alone treatment. Administrators could monitor not only minutes of activity but also exclusion, peer status, cooperation, and students’ perceived safety when intervening. Such recommendations are hypothesis-generating and theory-informed; intervention trials with longitudinal follow-up are required before claims of prevention effectiveness can be made.

### 4.5. Limitations and Future Research

First, all focal variables were measured in Wave 6, so temporal precedence was not established. Cross-sectional statistical indirect-association models can be biased when the underlying process is longitudinal and cannot distinguish forward, reverse, or reciprocal associations (Maxwell & Cole, 2007). Although Wave 6 was selected to focus on Grade 9 as the developmentally and educationally salient period immediately preceding the transition to high school and to compare the five concurrently available participant roles at the same stage, this rationale does not overcome the absence of temporal ordering. The findings, therefore, describe concurrent covariance patterns rather than developmental or causal mechanisms.

Second, several measurement limitations should be considered. Physical activity was represented by one ordinal self-report item about exercise sufficient to induce sweating during the previous week. Internal-consistency reliability is not applicable to this single item; test–retest reliability was not evaluated, and the measure omits exact frequency, intensity, duration, habitual patterns, activity type, and the social or instructional quality of the setting. All variables were also reported by the same adolescent at the same time, so social-desirability bias and common method variance remain possible. Harman’s single-factor test yielded eight components with eigenvalues greater than 1, and the first component accounted for 19.290% of the total variance; although no dominant single general factor was identified, this diagnostic cannot rule out common method bias. In addition, the documented five-subscale structure of the participant-role measure was retained and showed acceptable internal consistency, but its five-factor structure was not independently verified by confirmatory factor analysis in the present sample.

Third, limitations of model specification and statistical inference should temper interpretation. The inferential models controlled only for gender; urbanicity was reported descriptively, and socioeconomic resources, academic achievement or stress, school climate, parental involvement, prior bullying, and baseline mental health were not controlled. Residual confounding and omitted-variable bias may therefore account for part of the observed pattern. The five separate PROCESS models did not estimate residual covariances among the correlated outcomes, and the reported *p* values and bootstrap confidence intervals were not adjusted for multiplicity. Moreover, indirect estimates and model explanatory power were small, especially for defending and avoidance, and the positively skewed, low-frequency pro-bullying outcomes may be sensitive to distributional assumptions. The estimates should therefore not be interpreted as unconfounded or as strong evidence of practical importance.

Fourth, generalizability is restricted to South Korean Grade 9 adolescents assessed immediately before the transition to high school. Participant roles and their associations with physical activity may differ across age groups and educational systems because school-violence responses, peer norms, teacher practices, competitive climates, and opportunities for organized activity are context-dependent.

Future research should use appropriately spaced longitudinal designs with baseline adjustment; combine multi-informant, temporally separated, and objective measurements; include theoretically justified covariates and, where appropriate, school-level multilevel analysis; and apply distributionally appropriate or robust estimators.

Confirmatory studies should test the five-factor measurement structure and may use integrated structural equation models to estimate correlated participant roles and psychosocial variables jointly, together with multiplicity-adjusted inference. Measurement invariance should be evaluated when comparisons are made across gender, measurement occasions, or countries. Cross-national replication should also directly assess activity context, peer norms, moral disengagement, school climate, and students’ perceived safety in intervening.

## 5. Conclusions

This cross-sectional secondary analysis found that physical activity was concurrently associated with self-esteem, peer relationships, cooperativeness, and several bullying participant roles among South Korean ninth-grade adolescents. Separating five roles revealed different correlational patterns that would have been obscured by a single bullying score. This finding supports the value of understanding bullying participation not as a single undifferentiated behavior, but as a set of role-specific behaviors involving the initiation, assistance, reinforcement, defense, or avoidance of bullying situations.

Peer relationships and cooperativeness statistically accounted for small negative associations between physical activity and the three pro-bullying roles, whereas self-esteem accounted for a small positive association with defending. No psychosocial variable accounted for avoidance, and the defending and avoidance models had very low explanatory power. These role-specific patterns suggest that relational and behavioral resources may be more consistently associated with pro-bullying roles, whereas an intrapersonal resource such as self-esteem may be more selectively associated with active defending. However, these interpretations are based on small statistical associations observed at a single time point.

These results do not establish that physical activity or sport programs prevent bullying. They provide cautious, hypothesis-generating evidence that the social quality of activity settings may deserve attention alongside activity quantity, including features such as inclusion, cooperation, peer support, and safe opportunities to seek help. Longitudinal and intervention research is needed to determine temporal direction, practical magnitude, and whether inclusive, cooperative physical-activity contexts contribute to safer peer environments.

## Figures and Tables

**Table 1:** Demographic Characteristics of Participants.

Category	Group	Frequency (*n*)	Percentage (%)
Gender	Male	1128	50.3
	Female	1114	49.7
Urbanicity of school location	Metropolitan city	960	42.8
	Small- and medium-sized cities	925	41.3
	Rural town or township	357	15.9

**Table 2:** Descriptive Statistics, Normality, and Reliability for Major Variables.

Variable	*M*	*SD*	Skewness	Kurtosis	Cronbach’s *α*
Physical activity	2.400	1.330	0.616	−0.767	-
Self-esteem	2.883	0.419	0.319	0.106	0.792
Peer relationships	3.007	0.402	0.031	0.006	0.829
Cooperativeness	2.799	0.507	−0.321	0.325	0.930
Direct bullying	1.217	0.458	2.324	5.251	0.837
Assisting the bully	1.218	0.445	2.203	4.456	0.865
Reinforcing the bully	1.220	0.429	2.148	4.438	0.808
Defending the victim	1.682	0.776	0.898	−0.129	0.857
Avoidance of the bullying situation	1.750	0.855	0.863	−0.354	0.886

Note: Cronbach’s *α* was not calculated for physical activity because it was measured using a single item.

**Table 3:** Pearson Correlations among the Study Variables.

Variable	1	2	3	4	5	6	7	8	9	10
1. Physical activity	1									
2. Self-esteem	0.174 ***	1								
3. Peer relationships	0.196 ***	0.456 ***	1							
4. Cooperativeness	0.078 ***	0.169 ***	0.228 ***	1						
5. Direct bullying	−0.131 ***	−0.126 ***	−0.313 ***	−0.161 ***	1					
6. Assisting the bully	−0.132 ***	−0.142 ***	−0.325 ***	−0.159 ***	0.861 ***	1				
7. Reinforcing the bully	−0.091 ***	−0.116 ***	−0.274 ***	−0.156 ***	0.820 ***	0.825 ***	1			
8. Defending the victim	0.092 ***	0.093 ***	0.074 ***	0.026	0.223 ***	0.258 ***	0.267 ***	1		
9. Avoidance of the bullying situation	0.064 **	−0.024	0.001	−0.006	0.162 ***	0.187 ***	0.211 ***	0.565 ***	1	
10. Gender	−0.252 ***	−0.021	0.073 **	−0.005	0.001	0.007	−0.018	0.006	0.024	1

Note: Gender was coded as 0 = male and 1 = female. ** *p* < 0.01, *** *p* < 0.001.

**Table 4:** Concurrent Associations of Physical Activity with the Psychosocial Variables.

Outcome Variable	Predictor	*B*	*SE*	*β*	*t*	*p*	*LLCI*	*ULCI*	*R* ^2^
Self-esteem	Physical activity	0.057	0.007	0.181	8.390	<0.001	0.044	0.070	0.031
Peer relationships	Physical activity	0.070	0.006	0.230	10.822	<0.001	0.057	0.082	0.055
Cooperativeness	Physical activity	0.031	0.008	0.082	3.752	<0.001	0.015	0.047	0.006

Note: *B* = unstandardized regression coefficient; *SE* = standard error; *β* = standardized regression coefficient; *LLCI* = lower limit confidence interval; *ULCI* = upper limit confidence interval. All models controlled for gender. *R*^2^ indicates the proportion of variance explained by each regression model.

**Table 5:** Adjusted Concurrent Associations of Physical Activity and Psychosocial Variables with Bullying Participant Roles.

Outcome Variable	Predictor	*B*	*SE*	*β*	*t*	*p*	*LLCI*	*ULCI*	*R* ^2^
Direct bullying	Physical activity	−0.025	0.007	−0.072	−3.382	0.001	−0.039	−0.010	0.112
	Self-esteem	0.040	0.025	0.036	1.601	0.109	−0.009	0.088	
	Peer relationships	−0.335	0.026	−0.295	−12.734	<0.001	−0.386	−0.283	
	Cooperativeness	−0.085	0.019	−0.094	−4.584	<0.001	−0.122	−0.049	
Assisting the bully	Physical activity	−0.023	0.007	−0.068	−3.193	0.001	−0.036	−0.009	0.118
	Self-esteem	0.024	0.024	0.023	1.007	0.314	−0.023	0.071	
	Peer relationships	−0.335	0.025	−0.303	−13.152	<0.001	−0.385	−0.285	
	Cooperativeness	−0.077	0.018	−0.088	−4.300	<0.001	−0.113	−0.042	
Reinforcing the bully	Physical activity	−0.013	0.007	−0.041	−1.886	0.059	−0.027	0.001	0.086
	Self-esteem	0.024	0.023	0.023	1.010	0.313	−0.022	0.070	
	Peer relationships	−0.270	0.025	−0.253	−10.797	<0.001	−0.319	−0.221	
	Cooperativeness	−0.084	0.018	−0.099	−4.747	<0.001	−0.118	−0.049	
Defending the victim	Physical activity	0.048	0.013	0.082	3.682	<0.001	0.022	0.073	0.016
	Self-esteem	0.125	0.044	0.068	2.844	0.004	0.039	0.212	
	Peer relationships	0.046	0.047	0.024	0.989	0.323	−0.046	0.139	
	Cooperativeness	0.005	0.033	0.003	0.139	0.890	−0.060	0.070	
Avoidance of the bullying situation	Physical activity	0.053	0.014	0.082	3.650	<0.001	0.024	0.081	0.007
	Self-esteem	−0.074	0.049	−0.036	−1.523	0.128	−0.170	0.021	
	Peer relationships	−0.001	0.052	0.000	−0.015	0.988	−0.103	0.101	
	Cooperativeness	−0.010	0.037	−0.006	−0.278	0.781	−0.082	0.062	

Note: *B* = unstandardized regression coefficient; *SE* = standard error; *β* = standardized regression coefficient; *LLCI* = lower limit confidence interval; *ULCI* = upper limit confidence interval. All models controlled for gender. *R*^2^ indicates the proportion of variance explained by each regression model.

**Table 6:** Bootstrap Estimates of Statistical Indirect Associations between Physical Activity and Bullying Participant Roles.

Outcome Variable	Indirect Path	Indirect Estimate	BootSE	BootLLCI	BootULCI
Direct bullying	Physical activity → Self-esteem → Direct bullying	0.002	0.002	−0.001	0.006
	Physical activity → Peer relationships → Direct bullying	−0.023	0.003	−0.029	−0.017
	Physical activity → Cooperativeness → Direct bullying	−0.003	0.001	−0.005	−0.001
Assisting the bully	Physical activity → Self-esteem → Assisting the bully	0.001	0.002	−0.002	0.005
	Physical activity → Peer relationships → Assisting the bully	−0.023	0.003	−0.029	−0.018
	Physical activity → Cooperativeness → Assisting the bully	−0.002	0.001	−0.004	−0.001
Reinforcing the bully	Physical activity → Self-esteem → Reinforcing the bully	0.001	0.002	−0.002	0.005
	Physical activity → Peer relationships → Reinforcing the bully	−0.019	0.003	−0.024	−0.014
	Physical activity → Cooperativeness → Reinforcing the bully	−0.003	0.001	−0.005	−0.001
Defending the victim	Physical activity → Self-esteem → Defending the victim	0.007	0.003	0.002	0.013
	Physical activity → Peer relationships → Defending the victim	0.003	0.003	−0.003	0.010
	Physical activity → Cooperativeness → Defending the victim	0.000	0.001	−0.002	0.002
Avoidance of the bullying situation	Physical activity → Self-esteem → Avoidance of the bullying situation	−0.004	0.003	−0.011	0.002
	Physical activity → Peer relationships → Avoidance of the bullying situation	0.000	0.004	−0.008	0.008
	Physical activity → Cooperativeness → Avoidance of the bullying situation	0.000	0.001	−0.003	0.002

Note: BootSE = bootstrap standard error; BootLLCI and BootULCI = lower and upper limits of the 95% bootstrap confidence interval. Bootstrap samples = 5000. An interval excluding zero indicates a statistically detectable product of coefficients, not temporal or causal mediation.

## Data Availability

The data presented in this study are openly available from the National Youth Policy Institute (NYPI) Open Data Portal at https://www.nypi.re.kr/archive (accessed on 27 October 2025), under the title Korean Children & Youth Panel Survey 2018 (KCYPS 2018), Fourth-Grade Cohort, Wave 6 (2023). The dataset is publicly accessible in a de-identified format and can be downloaded upon registration and approval through the NYPI data request system.

## Supplementary Materials

The following supporting information can be downloaded at: https://www.mdpi.com/article/10.3390/bs16071192/s1, Table S1: KCYPS Items Used to Construct the Five Bullying Participant-Role Subscales.
