# Supplementary material for: Physical Activity and Bullying Participant Roles Among South Korean Adolescents: Parallel Statistical Indirect Associations Through Psychosocial Resources
Source: Behav Sci (Basel). 2026 Jul 15;16(7):1192. doi: 10.3390/bs16071192 (PMC13406104; doi:10.3390/bs16071192)
Supplement: Supplementary file 1 [file behavsci-16-01192-s001.zip › behavsci-4327322-supplementary.pdf]

Table S1. KCYPS Items Used to Construct the Five Bullying Participant-Role Subscales.

Role	Item	Item wording	Response scale
Direct bullying	1	I am the first to start bullying someone.	1 = never to 4 = often
	2	I get other students to join in bullying.	1 = never to 4 = often
	3	I am always looking for new ways to bully other students.	1 = never to 4 = often
Assisting the bully	1	When someone starts bullying another student, I join in.	1 = never to 4 = often
	2	I help the student who is bullying someone.	1 = never to 4 = often
	3	I help the bully in ways such as holding the victim so that the victim cannot get away.	1 = never to 4 = often
Reinforcing the bully	1	I go near the situation to watch the bullying.	1 = never to 4 = often
	2	I laugh while watching the bullying.	1 = never to 4 = often
	3	When someone bullies another student, I encourage the bully by saying things such as "Teach them a lesson."	1 = never to 4 = often
Defending the victim	1	I comfort the student being bullied or encourage them to tell a teacher.	1 = never to 4 = often
	2	I tell the students who are bullying to stop.	1 = never to 4 = often
	3	I try to stop the bullying.	1 = never to 4 = often
Avoidance	1	When someone is being bullied, I am usually not present.	1 = never to 4 = often
	2	I try not to become involved in bullying situations.	1 = never to 4 = often
	3	I do not take anyone's side in bullying situations.	1 = never to 4 = often

Note: English wording is a faithful translation of the Korean KCYPS items. Each role score was the mean of its three items. Source: National Youth Policy Institute (2024).
